# Laminar Profile of Auditory Steady-State Response in the Auditory Cortex of Awake Mice

**DOI:** 10.3389/fnsys.2021.636395

**Published:** 2021-03-19

**Authors:** Zijie Li, Jinhong Li, Shuai Wang, Xuejiao Wang, Jingyu Chen, Ling Qin

**Affiliations:** Department of Physiology, China Medical University, Shenyang, China

**Keywords:** EEG, gamma oscillation, auditory cortex, granular layer, thalamus

## Abstract

**Objective:**

Auditory steady-state response (ASSR) is a gamma oscillation evoked by periodic auditory stimuli, which is commonly used in clinical electroencephalographic examination to evaluate the neurological functions. Though it has been suggested that auditory cortex is the origin of ASSR, how the laminar architecture of the neocortex contributes to the ASSR recorded from the brain surface remains unclear.

**Methods:**

We used a 16-channel silicon probe to record the local field potential and the single-unit spike activity in the different layers of the auditory cortex of unanesthetized mice. Click-trains with a repetition rate at 40-Hz were present as sound stimuli to evoke ASSR.

**Results:**

We found that the LFPs of all cortical layers showed a stable ASSR synchronizing to the 40-Hz click stimuli, while the ASSR was strongest in the granular (thalamorecipient) layer. Furthermore, time-frequency analyses also revealed the strongest coherence between the signals recorded from the granular layer and pial surface.

**Conclusion:**

Our results reveal that the 40-Hz ASSR primarily shows the evoked gamma oscillation of thalamorecipient layers in the neocortex, and that the ASSR may be a biomarker to detect the cognitive deficits associated with impaired thalamo-cortical connection.

## Introduction

Recording the electrical activity of neurons, such as an electroencephalogram (EEG), is an effective means to evaluate the brain function. Recently, accumulating evidence suggests that auditory steady-state response (ASSR), measured non-invasively using an EEG, is an effective approach to evaluate the neural function in mental illness, including depression (Oda et al., 2012; Isomura et al., 2016; Zhou et al., 2018) and schizophrenia (Uhlhaas and Singer, 2010; Thuné et al., 2016), and in neuropharmacological experiments on animal models (Leishman et al., 2015; Shahriari et al., 2016; Sivarao et al., 2016). The ASSR is an electrophysiological response entrained to both the frequency and the phase of rapid, periodic auditory stimuli (Galambos et al., 1981; Brenner et al., 2009). EEG measurement of ASSR in the gamma frequency range (40-Hz) is often assumed to reflect the integrity of the sensory pathways and the capacity of these pathways to generate synchronous activity.

The physiological processes generating ASSR are still being studied. Many results of magnetoencephalography (MEG) studies on human subjects have suggested that the auditory cortex (AC) may be a source of ASSR (Herdman et al., 2003; Teale et al., 2008; Lazzouni et al., 2010; Kuriki et al., 2013; Bharadwaj et al., 2014). This is also supported by some electrophysiological recording results from animal experiments (Wang et al., 2019). It should be noted that one of the most prominent features of the mammalian neocortex is the laminar architecture in cortical information processing. It has been shown that the primary thalamic afferents, which drive the sensory responses, mainly projects toward the granular layers of the AC (Romanski and LeDoux, 1993; Kimura et al., 2003; Winer and Lee, 2007). In contrast, the supra- and infragranular layers do not receive the same proportion of inputs from the thalamus, but mediate the corticocortical connections for the feedback and feedforward pathways (Felleman and Van Essen, 1991). Previous electrophysiological studies on rodents have found that the neurons in the different layers of the AC exhibited different spontaneous and sensory-evoked activities, showing differences in the flow of information through cortical circuits (Rouiller et al., 1991; Hromádka et al., 2008; Sakata and Harris, 2009), and revealed differential involvements of supragranular, granular, and infragranular layers in auditory attentional processes (Ramayya et al., 2014; De Martino et al., 2015; Francis et al., 2018) and multisensory interactions (Lakatos et al., 2007). There have been many studies on the laminar profiles of electrical rhythm in the sensory regions of the neocortex (Lakatos et al., 2005; Haegens et al., 2015; Sotero et al., 2015; Senzai et al., 2019). However, the role of laminar architecture of the AC in the origin of ASSR remains elusive. To reveal the origin of ASSR from the layer-dependent neural activities, here, we analyzed the laminar multielectrode data recorded in the AC of unanesthetized mice to explore how the local field potential (LFP) and the single-unit spike activity (SUA) in different cortical layers contributes to the ASSR recorded at the pial surface. We found a non-uniform distribution of neural response evoked by a 40-Hz click-train across the layers of the AC, and the strongest generators of ASSR are in the granular cortical layers.

## Materials and Methods

### Animals

All experiments were performed in adult female (*n* = 8) KM mice between 6 and 8 weeks of age weighing between 18–25 g. Mice were housed on a 12 h/12 h day/night cycle. Mice were free to eat and drink. All procedures were approved by the Animal Ethics Committee (CEUA) of China Medical University (No. KT2018060). All surgeries were performed under anesthesia, and all efforts were made to minimize animal suffering.

### Surgical Procedures

Mice were anesthetized with isoflurane in conjunction with air (3% for induction and 1–2% for maintenance) and fixed in a stereotaxic apparatus with blunt ear bars (#68001, RWD Life science, Shenzhen, China). After shaving the hair, to expose the skull, an incision was made and the skull surface was cleaned with hydrogen peroxide solution (5%) and dried off with an air puffer. The skull was positioned such that the lambda and bregma marks were aligned on the anteroposterior and dorsoventral axes. A custom-designed headpost was attached to the skull with four screws for head fixation. The headpost was fixed with dental cement for later recording. Mice recovered 2 weeks before the experiments began. Analgesic was given once before and for 3 days following surgery.

### Electrophysiological Recording

**FIGURE 1:**
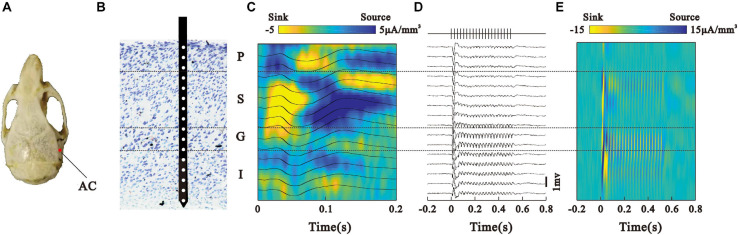
Laminar bistability profile of LFPs in the AC. **(A)** Projection point of the AC on the skull. **(B)** Laminar structure of cortex and the recording electrode location. **(C)** Noise-evoked LFPs (black line) recorded from different sites in the AC and average current source density (CSD) map. **(D)** LFPs in response to the 40-Hz click-train stimulus. Vertical lines at the top represent the click-train stimulus. **(E)** CSD map constructed by the LFPs in panel **(D)**.

After the animals recovered from surgery, they were habituated with the experimental devices in a sound-attenuated recording room. The mice were head-fixed on a custom-made frame through the headpost. The body rested atop a disk, which was mounted on a low-friction, silent rotor. This habituation procedure lasted for 30 min per day and was repeated for 3 days. On the fourth day, the mice were briefly anesthetized with isoflurane, and a 500 μm diameter craniotomy was performed on top of the AC (AP −3.0 mm; ML −4.0 mm; DV 2.0 mm, Figure 1A). The dura was removed and the craniotomy was protected with saline. After the animal completely recovered from anesthesia, a single-shank linear silicon electrode with 16 recording sites spacing regularly at a 50 um distance (Neuro-Nexus, A1 × 16–3.8 mm-50-177) was inserted into the AC perpendicular to the brain surface. Ground and reference electrodes were positioned on the interparietal bone. The electrode was mounted on a remotely controlled manipulator (MO-10, Narishige, Japan) and gradually penetrated into the cortex under audio-visual monitoring the recorded electrophysiological signals. The penetration was held after the top recording site just entered the brain tissue (Figure 1B), indicated by an abrupt change of a potential wave pattern. After determining the laminar structure using a noise sound stimulus, the recording experiment of a click-train stimulus started. At the end of the recording session, a small chamber was built around the craniotomy with cement and filled with ointment. The chamber was removed before each subsequent recording session and rebuilt after it. Typically, each animal was recorded for four to seven sessions.

### Acoustic Stimuli

When the silicon probe reached an AC column, broadband noise bursts were used to evoke the CSD pattern to find the laminar position. The noise was 80 ms in duration, 70 dB, 75 trials in one session, and 1.5 s interval. We used a train of click sounds as sound stimulus to evaluate ASSR. One click of the click-train was a 0.2 ms duration rectangular pulse. The click-train lasted for 0.5 s and repeated for 40 cycles/s. MATLAB (Mathworks, United States) was used to digitally generate 100 kHz of sampling rate waveforms. A D/A board (PCI-6052E, National Instruments, United States) was used to transfer the waveform to an analog signal, and a loudspeaker (K701, AKG, Vienna, Austria) was used to play it. The click-train intensity was adjusted to 70 dB SPL and measured at the middle of the recording box (Brüuel and Kjaer type 2,238 sound level meter, Naerum, Danish). In one session, 120 trials of click-train were played at a random interval between 4 and 8 s.

### Acquisition and Analysis of Electrophysiological Data

The microwire output was delivered to a multi-channel preamplifier (RA16PA; TDT, Alachua, FL, United States), then to a digital signal processing module (RZ-5; TDT). The signal was amplified and split into LFP (0.1–300 Hz) and SUA (300–5,000 Hz) range by filtering. The data was imported into the MATLAB for analysis. The current-source density (CSD) was calculated as the second spatial derivative of the LFP signal according to the following formula:

CSD=(V-h-Δh2V+hV)h+Δh/Δh2

Where *V* is the potential at position *h* and Δ*h* is the distance between the electrodes. CSD represents the net current density entering or leaving the extracellular medium at a particular spatial position. Noise-evoked columnar CSD patterns were used to determine the layer of the AC. Ten milliseconds after the sound started, a brief current sink appears (Figure 1C). The granular layer of the AC was determined by this biomarker (Kaur et al., 2005; Leszczyński et al., 2020). Normally, three channels were assigned to the granular layers. The position of the granular layer was used to identify the positions of the supragranular and infragranular layers.

We used a custom-written Matlab script to analyze the LFPs evoked by the click-trains at 40 Hz. The 120 trials of LFP recorded in one session were analyzed by a wavelet-based analysis algorithm embedded in the eeglab toolbox^1^ to calculate the mean trial power (MTP) and phase-locking factor (PLF) (Delorme and Makeig, 2004). The MTP is obtained by calculating the power distribution in the spectral-temporal domain for each trial, and then constructing a mean spectral-temporal function averaged across one recording session. The PLF in one session described the phase synchronization across individual trials of LFP at particular frequencies and time intervals. For the data recorded from one session, the MTP and PLF were calculated to obtain a spectral-temporal function using the multitaper method provided by the eeglab. The results presented as a relative estimation of the ratio between the values after stimulus onset and baseline value (stimulus/pre-stimulus). We also calculated the evoked power by subtracting the non-phase locking compartments (induced power) from the total power (Steinschneider et al., 2008).

All spike detection and sorting took place off-line using the OpenSorter software (TDT). Peristimulus time histogram (PSTH) of each trial was constructed by calculating the instantaneous firing rate (1 ms bin) from SUA for each stimulus presentation from 500 ms pre- to 1,000 ms post-stimulus-onset. Then, PSTH was smoothened with a 5 ms Gaussian sliding window.

### Neural Contribution to the ASSR of Pial Surface

Referring to the anatomical characteristics of the AC (Hackett et al., 2001), we used the LFP signal recorded from the top electrode channel to represent the signal at the pial surface of the AC. We calculated the coherence between the LFPs of pial and deeper layers by using the eeglab toolbox function “newcross” to research which laminar LFP is the origin of the pial surface ASSR. Event-related phase coherence (ERCOH) is a phase coupling factor between the two signals. The ERCOH result is a spectral-temporal function and the values are between zero (0) and one (1). The value indicates the phase and amplitude level of coherence between the two signals.

Granger causality was used to examine the flow of information between the cortical layers. Granger causality is based on the concept of time series prediction. For two simultaneously measured time series, one series can be called causal to the other if we can better predict the second series by incorporating past knowledge of the first one. Granger causality has been applied to reveal the generator of alpha current in a monkey’s visual cortex (Bollimunta et al., 2008, 2011). We conducted Granger causality analysis on the CSDs obtained from the different cortical layers. To test whether the Granger causality value is significantly greater than zero, a random permutation approach was adopted (Bollimunta et al., 2008). We randomly shuffled trial indices to create a synthetic ensemble of trials. Such random permutations were repeated many times, resulting in a distribution of causality value that corresponds to the null hypothesis of no statistical interdependence. The causality value calculated from the actual data is compared with this baseline null hypothesis distribution for the assessment of significance levels (*p* < 0.01).

### Phase-Locking of SUA to LFP

We also calculated the phase-locking between the pia LFP and SUAs recorded at the different layers by using the spike-field coherence (SFC) analysis. SFC is commonly used to compute phase synchronization between spikes LFPs (Gregoriou et al., 2009; Jutras et al., 2009; Chalk et al., 2010). All LFPs from each layer group were averaged to rule out the possible contamination of spikes (Waldert et al., 2013). The cohgramcpt function (nine tapers, five time-band width) of the Chronux toolbox was used to obtain the power spectra of PSTH and LFP. Then, we computed the coherence between spikes and LFPs using the formula below (Fries et al., 2001; Jarvis and Mitra, 2001).

S_SL_ indicates the cross-spectrum between spikes and LFPs. S_S_ and S_L_ indicate the autospectra of spikes and LFPs, respectively. If there is no phase synchronization between spikes and LFPs, the value of C_SL_ will be zero. C_SL_ will be one if they are perfectly synchronized.

### Histology

We confirm the position and depth of the electrode at the end of the experiment. Electrodes were confirmed by microscopic observation. All electrodes were implanted into correct positions.

### Statistical Analysis

Kruskal–Wallis test was performed on the comparisons between the data of the different layers. Each Kruskal–Wallis test reporting significant effects was followed by the Tukey’s *post hoc* test of multiple comparison.

## Results

### Laminar Profile of LFPs Evoked by 40-Hz Click-Trains

We investigated the neural activities generating ASSR by analyzing the LFP and accompanying SUA signals in the mouse’s AC (eight animals, 42 experimental sessions) recorded with laminar multi electrodes. Figure 1A shows the electrode location in the skull. We firstly used the CSD responses to noise sounds to localize the laminar profile of cortical LFPs (Figures 1B,C), then analyzed their spatiotemporal relationship to the ASSR recorded at the pial surface, and determined the neuronal generators of ASSR in the AC. Consistent with earlier work (Sakata and Harris, 2009; Guo et al., 2017; Leszczyński et al., 2020), we localized the LFPs into four groups: pial surface (P), supragranular (S), granular (G), and infragranular (I) layer. Next, we present the representative LFPs averaged across 120 click-train stimulus trials in one session (Figure 1D). The LFPs showed a larger deflection at the onset of stimulation, and a train of adapted response synchronizing to the stimulus frequency (40-Hz) during the steady state of stimulation. We also constructed a laminar map of CSD driven by click-train stimulus, which showed strong responses in the G and S layers (Figure 1E).

**FIGURE 2:**
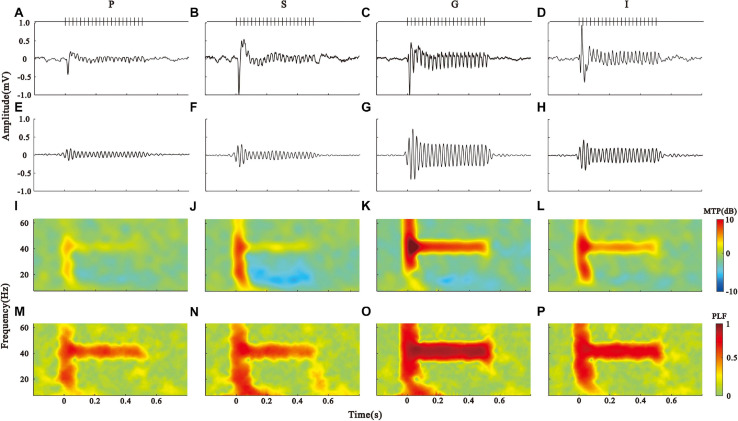
Layer-specific ASSR patterns in the AC. **(A–D)** Click-train evoked LFPs averaged across the pia (P), supragranular (S), granular (G), and infragranular (I) layers, respectively. **(E–H)** 35–45 Hz filtered LFPs. **(I–L)** spectral-temporal function of MTP in the P, S, G, and I layers. **(M–P)** spectral-temporal function of PLF in the P, S, G, and I layers.

To conduct a quantitative comparison between the layers, we divided the LFPs into four groups according to the CSD profile and plotted the mean LFPs averaged across each group in Figures 2A–D. To compare the LFP oscillation with the stimulus frequency, the LFPs were further filtered through a linear-phase FIR bandpass filter of 35 − 45 Hz (Figures 2E–H). This process was fulfilled using the “eegfilt” function in the EEGLAB toolbox, which caused a little shifting of signal onset in the temporal domain. All groups of LFP displayed a big fluctuate at the onset of stimulus, followed by a clear oscillation throughout the stimulus period (ASSR), while the response amplitude was largest in the G layer.

**FIGURE 3:**
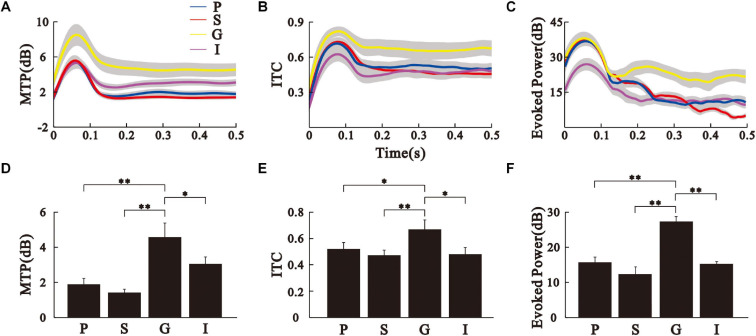
Comparison of MTP, PLF, and evoked power between the different AC layers. **(A–C)** time function of MTP, PLF, and evoked power of the different AC layers averaged over eight animals. Sold line represents mean, shade area represents SE. **(D–F)** Bar graph shows the mean and SE of MTP, PLF, and evoked power values averaged across the 35–45 Hz frequency and 0.2–0.5 s post-stimulus time window. ^∗∗^*p* < 0.01, ^∗^*p* < 0.05, Kruskal–Wallis test followed by Tukey’s multiple comparison.

To assess the ASSR, we conducted the spectrotemporal analysis of MTP (Figures 2I–L) and PLF (Figures 2M–P) on the LFPs. The population results of MTP and PLF are presented by the time function of MTP and PLF averaged across the 35–45 Hz frequency range (Figures 3A,B). These results were further quantified by calculating the 40-Hz MTP and PLF values averaged across the 35–45 Hz frequency and 0.2–0.5 s post-stimulus time window. The MTP and PLF locking to the stimulus frequency (40 Hz) were significantly higher in the G layer than those in other layers (Figures 3D,E, Kruskal–Wallis test, MTP: *F*_(3,164)_ = 77.30, ITC: *F*_(3,164)_ = 22.93, ^∗∗^*p* < 0.01, ^∗^*p* < 0.05, Tukey’s multiple comparison). We also calculated the evoked power of ASSR to remove the induced power of a non-phase locking response. The differences of evoked power between the layers were consistent with that of MTP (Figures 3C,F, Kruskal–Wallis test, *F*_(3,164)_ = 28.28, ^∗∗^*p* < 0.01, Tukey’s multiple comparison). These results suggest that the G layer is the origin of the 40-Hz ASSR in the AC.

### Coherence Between the LFPs of Pial Surface and Deep Layers

**FIGURE 4:**
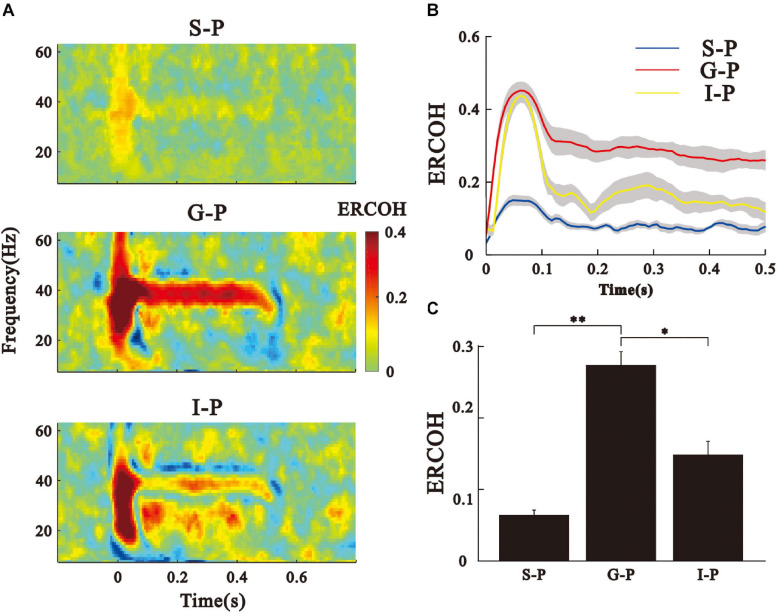
ERCOH between the LFPs of pial surface and deep layers. **(A)** Spectral-temporal function of ERCOH between the P layer and other layers. **(B)** Time function of ERCOH (35–45 Hz) of the different pairs of layers averaged over eight animals. Sold line represents mean, shade area represents SE. **(C)** Bar graph shows the mean and SE of ERCOH values averaged across the 35–45 Hz frequency and 0.2–0.5 s post-stimulus time window. ^∗∗^*p* < 0.01, ^∗^*p* < 0.05, Kruskal–Wallis test followed by Tukey’s multiple comparison.

Next, we examined the coherence between the LFPs of the different layers (ERCOH) to reveal the potential source of pial ASSR. An example for one session and one animal of the ERCOH spectrum (between 0 and 60 Hz) averaged over trials was shown (Figure 4A). We observed an obvious increase of ERCOH at 40-Hz between the G and P layers, and between the I and P layers during the steady post-stimulus period. The consequences of 40-Hz coherences averaged over sessions and animals were shown in mean and SE (Figure 4B). Figure 4C displays that the mean ERCOH (35–45 Hz frequency range, 0.2–0.5 s post-stimulus time window) between the P and G layers was significantly higher than the other two pairs [Kruskal–Wallis, *F*_(2,123)_ = 89.11, ^∗∗^ *p* < 0.01, ^∗^*p* < 0.05, Tukey’s multiple comparison].

### Laminar Profile of CSD

**FIGURE 5:**
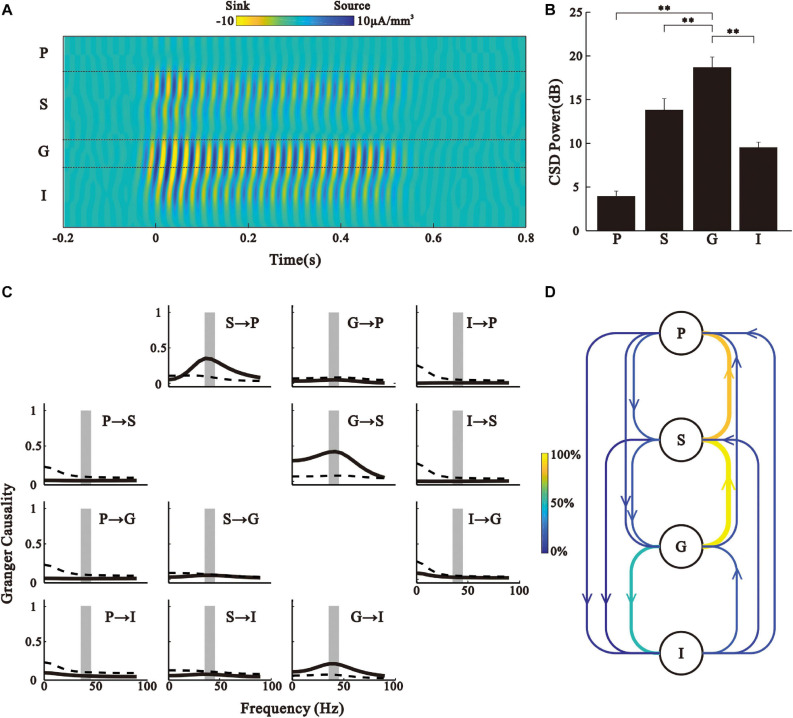
CSD analysis. **(A)** Laminar map of 35–45 Hz CSD driven by 40 Hz click-train for the example in Figure 1. **(B)** Bar graph shows the mean and SE of CSD power during 0.2–0.5 s post-stimulus time window. ^∗∗^*p* < 0.01, Kruskal–Wallis test followed by Tukey’s multiple comparison. **(C)** Granger causality spectra for different layer pairs for the example in Figure 1. The *y*-axis is the target and *x*-axis is the driver (i.e., panel *xy*, where *x* is the row index of the panel and *y* is the column index, shows the Granger causality spectrum for *x*- > y). Solid line is the Granger causality spectrum. Dashed line shows the threshold level of statistical significance (*p* < 0.01, random permutation approach). Vertical shaded strip marks the frequency band of 35–45 Hz. **(D)** Schematic representation of directional information flow between different layers. Color of an arrow represents the percentage of significant Granger causality observed in all the recorded trials.

To exclude the potential effect of volume conduction on LFP, we analyzed the CSD of 40 Hz ASSR. Figure 5A shows the laminar profile of CSD of the representative recording. The CSDs were calculated from the 35 − 45 Hz filtered LFPs, similar to those shown in Figures 2E–H. Consistent with the results based on the LFP signals, 40 Hz CSD was strongest in the G layer, weakest in the P layer, and intermediate in the S and I layers (Figure 5B, Kruskal–Wallis, *F*_(3,164)_ = 40.67, ^∗∗^*p* < 0.01, Tukey’s multiple comparison).

We further used Granger causality analysis to examine directional causal influences between the CSDs of the different layers, and found a significant peak at the spectrum of Granger causality around 40 Hz in the direction of G- > S, G- > I, and S- > P (Figure 5C). For the population analysis results of each layer pair, we counted the percentage of occurrence of a significant Granger causality peak in the 35 − 45 Hz range to represent the strength of directional information flow (Figure 5D). These results further suggest that the G layer is the generator of ASSR in the AC.

### Phase-Locking of SUA to the LFP of Pial Surface

**FIGURE 6:**
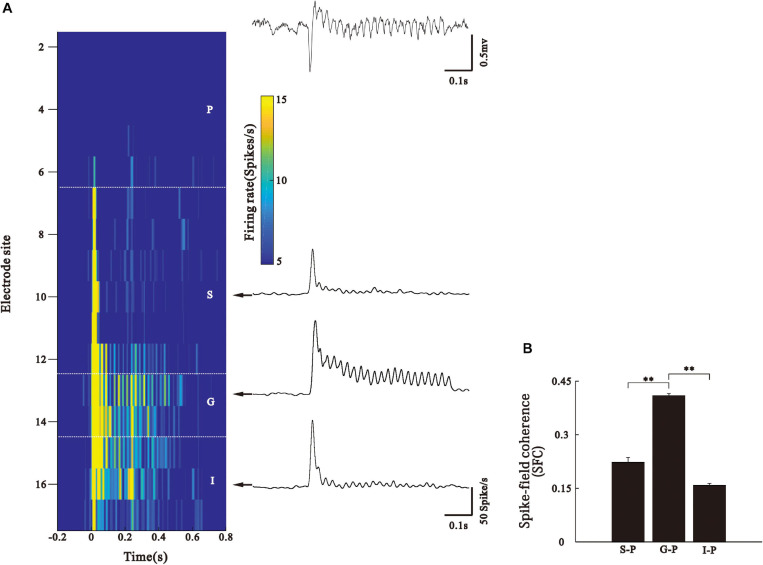
Phase-locking of SUA to the LFP of pial surface. **(A)** Left: firing rate of SUA (represented by color) simultaneously recorded from different layers. Right: LFP recorded at pial surface and representative PSTH in each layer. **(B)** Bar graph shows mean and SE of SFC values averaged across the 35–45 Hz frequency and 0.2–0.5 s post-stimulus time window. ^∗∗^*p* < 0.01, Kruskal–Wallis test followed by Tukey’s multiple comparison.

We also used SFC to investigate synchronization between the neuronal activities of different cortical layers and the pial ASSR. The examples of SUA recorded from the different layers of AC in one electrode penetration (Figure 6A). The firing rate of neurons in the S layer demonstrated a transient increase at the stimulus onset, which rapidly flowed to the background level during the steady period of stimulus. In contrast, neurons in the G layer could periodically fire synchronizing to the stimuli. Neurons in the I layer exhibited a discharge pattern intermediating between those of the S and G layers. For each SUA, we conducted a SFC analysis with the simultaneously recorded LFP in the P layer. This analysis confirmed that the strongest 40-Hz phase locking during the stimulus period occurred between the SUA of the G layer and LFP of the P layer (Figure 6B, Kruskal–Wallis, *F*_(2,273)_ = 201.17, ^∗∗^*p* < 0.01, Tukey’s multiple comparison). It should be notified that the SUA and LFP signals were obtained through the band pass filters with different frequency bands, therefore, SUAs cannot directly contribute to LFP. The coherence between them only suggests that the ASSR of LFP recorded in the pial layer correlated with the SUAs in the G layer.

## Discussion

Gamma oscillations are well-known features of cortical activity, which may play an important role in local gain control (Cardin, 2016; Sohal, 2016) and signal transmission among cortical networks (Akam and Kullmann, 2010; Fries, 2015). Though the mechanism of gamma oscillations still is not entirely clear (Butler and Paulsen, 2014; Bastos et al., 2015; Ray and Maunsell, 2015; Lasztóczi and Klausberger, 2016; Sohal, 2016), alterations in gamma oscillations have been used as a biomarker of cortical functions (Herrmann and Demiralp, 2005; Uhlhaas and Singer, 2006, 2010; Basar-Eroglu et al., 2007; Antonoudiou et al., 2020). Forty Hertz (40-Hz) ASSR is a gamma oscillation evoked by auditory stimuli, which is commonly measured with the use of an EEG in the clinic to evaluate the neurological functions. Uncovering the neural source of 40-Hz ASSR is helpful to close the gap between human neurological manifestations and animal electrophysiology consequences. Thus, it is necessary to investigate the correlation between ASSR of the EEG recorded from the brain surface and the neural electric signals recorded inside the cortex. In this study, we compared the laminar structure of CSD, LFP, and SUA evoked by 40-Hz click-trains in the AC. We found that the neural activity showed the strongest synchrony in presumptive thalamorecipient layers (G layers), and the ASSR recorded in the pial surface was the most correlative with the activation of thalamorecipient layers.

Previous anatomical and physiological evidence shows that auditory responses in the AC originate mainly from the thalamic afferents, which is biased toward the granule layers (Romanski and LeDoux, 1993; Kimura et al., 2003; Winer and Lee, 2007). Consistent with this, 40-Hz click-trains evoked the strongest activation in putative thalamorecipient layers (G layers), from where spreading to the S and I layers. It has been shown that the S and I layers mediate corticocortical connections (Felleman and Van Essen, 1991), which are involved in attentional processes (O’Connell et al., 2014; De Martino et al., 2015; Francis et al., 2018) and multisensory interactions (Lakatos et al., 2007). Our results show that the strength of ASSR in the S and I layers was significantly lower than that in the G layers, indicating that the rhythmic thalamocortical signals are inhibited as transmission in the cortex. Functional connectivity with higher-order cortical areas may contribute to the inhibition of ASSR transmission. For example, top-down projections from the prefrontal cortex have been found to target neurons in the S layer in the AC (Jones et al., 2011; Medalla and Barbas, 2014; Plakke and Romanski, 2014). Future studies measuring ASSR simultaneously in laminar profiles of the AC and higher-order cortex will help to clarify the cortico-cortical network dynamics of ASSR associating with sensory behavioral. Furthermore, there are also other possible reasons; for example, the inhibitory input from the G layer to the S layer within the cortical column may be faster than the excitatory input, which reduces the responsibility of S layer neurons to a click-train stimulus. Several studies on the somatosensory cortex have shown that both parvalbumin-expressing (PV+) interneurons and pyramidal neurons in the thalamorecipient layers receive direct thalamic inputs, but PV+ interneurons respond faster than pyramidal neurons (Sermet et al., 2019; Yu et al., 2019). Thus, the cell-type-specific study is necessary to reveal the intracortical regulation mechanism of ASSR.

It has been suggested that the generation of gamma rhythms depends on the inhibitory interneurons spiking. The fast synaptic inhibition inhibiting the firing of excitatory pyramidal cells is one reason (Bartos et al., 2007; Buzsáki and Wang, 2012; Kim et al., 2016; Chen et al., 2017; Veit et al., 2017). PV+ interneurons target the perisomatic domain of pyramidal neurons. In the brain, it is important for gamma oscillations generating and maintaining (Hájos and Paulsen, 2009; Tukker et al., 2013; Cardin, 2016). PV+ interneurons are adapted for fast synchronization of network activity, as they resonate at gamma frequencies and exert strong perisomatic inhibition that is capable of precisely controlling spike timing (Cardin et al., 2009; Hu et al., 2014; Kohus et al., 2016). In addition, dendrite-targeting somatostatin-expressing (SST +) interneurons are also suggested to contribute to the generation of gamma oscillation (Chen et al., 2017; Veit et al., 2017; Hakim et al., 2018; Antonoudiou et al., 2020). Studies on the rodent somatosensory cortex found that SST + interneurons can provide differential inhibition in a cortical layer specific way to excitatory neurons (Xu et al., 2013). How the SST and PV sub-system cooperate to influence the dynamics of gamma oscillations remains unclear. Consider that PV neurons are frequently found in thalamorecipient layers and have large, fast synaptic conductance and short membrane time-constants, they might be the main contributor to the early and strong CSD of ASSR observed in the G layer. Direct thalamic input to SST neurons is weak (Cruikshank et al., 2010; Audette et al., 2018). SST neurons receive intracortical inputs from both pyramidal and inhibitory neurons (Yu et al., 2019). The rapid and large input from PV neurons may delay and reduce the response of SST neurons, which perhaps contribute to the CSD of ASSR in the S and G layers. These speculations require being examined in the future using cell-type-specific methods.

In conclusion, we found that 40-Hz ASSR primarily reflects the evoked gamma oscillation of thalamorecipient layers in the neocortex. Accumulating evidence suggests that the defects in the thalamo-cortical pathway are associated with schizophrenia (Woodward et al., 2012; Giraldo-Chica et al., 2018; Kupferschmidt and Gordon, 2018), and aberrant gamma oscillations are also consistently observed in the studies with schizophrenia patients (Ferrarelli et al., 2008; Gonzalez-Burgos and Lewis, 2008; Uhlhaas and Singer, 2010; Jadi et al., 2016; Zhou et al., 2018). Therefore, our current finding suggests that ASSR of EEG recording can be used as a biomarker to examine the function of thalamo-cortical circuit and detect the cognitive deficits associated with impaired gamma oscillation.

## Data Availability Statement

The raw data supporting the conclusions of this article will be made available by the authors, without undue reservation.

## Ethics Statement

The animal study was reviewed and approved by Animal Ethics Committee (CEUA) of China Medical University.

## Author Contributions

ZL was primarily responsible for experiment studies and statistical analysis. SW, JL, JC, and XW assisted with statistical collection and analysis. LQ was primarily responsible for statistical analysis and manuscript editing. All authors contributed to and approved of the final version of the manuscript.

## Conflict of Interest

The authors declare that the research was conducted in the absence of any commercial or financial relationships that could be construed as a potential conflict of interest.

## Footnotes

1 https://sccn.ucsd.edu/eeglab/index.php
